# Estimation of Lower Limb Joint Angles and Joint Moments during Different Locomotive Activities Using the Inertial Measurement Units and a Hybrid Deep Learning Model

**DOI:** 10.3390/s23229039

**Published:** 2023-11-08

**Authors:** Fanjie Wang, Wenqi Liang, Hafiz Muhammad Rehan Afzal, Ao Fan, Wenjiong Li, Xiaoqian Dai, Shujuan Liu, Yiwei Hu, Zhili Li, Pengfei Yang

**Affiliations:** 1 Key Laboratory for Space Bioscience and Biotechnology, School of Life Sciences, Northwestern Polytechnical University, Xi’an 710072, China; wangfanjie@mail.nwpu.edu.cn (F.W.); liangwenqi@mail.nwpu.edu.cn (W.L.); rehan@nwpu.edu.cn (H.M.R.A.); fa_study@mail.nwpu.edu.cn (A.F.); yiwei.hu@nwpu.edu.cn (Y.H.); 2 National Key Laboratory of Space Medicine, China Astronaut Research and Training Center, Beijing 100094, China; muzijiong2007@163.com (W.L.); daixiaoqian1985@163.com (X.D.); mao6681467@163.com (S.L.)

**Keywords:** joint angle estimation, joint moment estimation, deep learning, inertial measurement unit

## Abstract

Using inertial measurement units (IMUs) to estimate lower limb joint kinematics and kinetics can provide valuable information for disease diagnosis and rehabilitation assessment. To estimate gait parameters using IMUs, model-based filtering approaches have been proposed, such as the Kalman filter and complementary filter. However, these methods require special calibration and alignment of IMUs. The development of deep learning algorithms has facilitated the application of IMUs in biomechanics as it does not require particular calibration and alignment procedures of IMUs in use. To estimate hip/knee/ankle joint angles and moments in the sagittal plane, a subject-independent temporal convolutional neural network-bidirectional long short-term memory network (TCN-BiLSTM) model was proposed using three IMUs. A public benchmark dataset containing the most representative locomotive activities in daily life was used to train and evaluate the TCN-BiLSTM model. The mean Pearson correlation coefficient of joint angles and moments estimated by the proposed model reached 0.92 and 0.87, respectively. This indicates that the TCN-BiLSTM model can effectively estimate joint angles and moments in multiple scenarios, demonstrating its potential for application in clinical and daily life scenarios.

## 1. Introduction

Analysis of lower limb joint kinematics and kinetics is essential for diagnosing gait disorders and assessing the rehabilitation process [1,2]. Therefore, how to accurately and conveniently obtain joint angles and moments has always been a popular research topic.

Conventional gait analysis techniques rely on specialized biomechanical laboratories with optical motion capture systems and force plates. Joint angles and moments can be calculated by personalized musculoskeletal models combined with inverse kinematics and inverse dynamics methods. Although this approach is considered as the gold standard for dynamic analysis of joint angles/moments, it requires expensive specialized equipment and complex manual data processing, which means that the method can only be applied within limited scenarios [3]. To overcome some of the aforementioned limitations, inertial measurement units (IMUs) were introduced for joint kinematics [4,5] and kinetic [6,7] analyses. It has been suggested that joint angles and moments can be calculated using computational models [8] or musculoskeletal models [9] combined with motion information from IMUs. However, most model-based methods for calculating joint angles and moments require complex setup processes for the IMUs before collecting motion data. For example, the IMUs are supposed to be aligned with each specific limb segment and calibrated [10,11]. Although several alignment [12,13] and calibration [14,15] methods have been proposed, the process of alignment and calibration may be prone to errors because they are challenging to implement onto subjects consistently. Since the majority of model-based methods for estimating joint angles and moments cannot avoid the complex setup processes of IMUs, it makes them too cumbersome to be applied in daily monitoring.

The excellent performance of deep learning models combined with IMU data in estimating joint kinematics and kinetics [16,17] has attracted great attention. Deep learning models minimize the static pose calibration procedure and cumbersome sensor setup, as they can extract relevant features from the raw data of IMU sensors to estimate joint angles and moments. In addition, data-driven deep learning models can avoid the complexity of biomechanical modeling and the dependency on magnetometer data [16].

To select suitable deep learning models for gait analysis outside the specialized biomechanics laboratory environment, the performance of three commonly employed deep learning methods in estimating both joint kinematics and kinetics based on IMU data was compared. The results revealed that convolutional neural networks (CNN) can achieve the highest prediction accuracy, but they demand a comprehensive dataset and complex preprocessing process. Although the performance of the long short-term memory (LSTM) network was inferior, it is more conducive to real-time applications [18]. In subsequent research [19], the LSTM recurrent neural network was proposed to estimate multi-joint angles based on a low-frequency IMU sensor. The results suggested that the joint angle coefficient of determination estimated by the LSTM model was greater than 0.74 compared with the optical motion capture system. Likewise, the modular LSTM model and wearable IMUs were applied to predict the external knee extension moment in real time. The estimation accuracy of the external knee extension moment during different drop landing ways was R^2^ = 0.84 ± 0.14 and R^2^ = 0.84 ± 0.12, respectively [20]. The artificial neural network (ANN) has also been applied to predict lower limb joint angles and moments using a single IMU fixed to the sacrum. The ANN prediction results indicate that the average errors of segment angles range from 2.2° to 3.4°, and the approximate errors of lower limb joint moments range from 11.4 Nm to 16.7 Nm [21]. However, the deep learning methods described above still have some limitations in estimating joint kinematics and kinetics during different locomotive activities. To achieve joint moment estimation during four different movement patterns, a new deep learning model DL-Kinetics-FM-Net based on a single foot IMU sensor was created. Compared to the feedforward neural network (FNN), the DL-Kinetics-FM-Net resulted in a decreasing NRMSE by 7.10–23.16% for joint moment estimation [17].

Since data-driven deep learning methods require a comprehensive and representative training dataset, simulated IMU data synthesized based on optical motion capture data were introduced to expand the training dataset [16,22]. In previous research, the FNN model and synthetic simulated IMU data were used to predict joint angles and moments, and the prediction results suggested that the average correlation coefficients of joint angles and joint moments were 0.85 and 0.95, respectively [23]. In another study, synthetic IMU data related to kinematic prediction were generated using musculoskeletal modeling software to augment the dataset. Synthetic IMU data are different from simulated IMU data; they are the artificial IMU data generated by the motion beyond the experimental observation. Using these synthetic IMU data to train the neural networks improved the accuracy of hip, knee, and ankle joint kinematic prediction [24]. Since simulated and synthetic IMU data do not include any vibration noise, these non-realistic IMU data may not provide realistic and reliable kinematic and kinetic estimation in practical applications.

To overcome the limitations imposed by musculoskeletal model modeling, static calibration of the IMU, simulated IMU data, and synthetic IMU data, this study aims to develop a novel deep neural network to estimate lower limb kinematics and kinetics using three IMUs during different locomotive activities, including level-ground walking, ramp ascent/descent, stair ascent/descent, and treadmill walking. To achieve this purpose, a temporal CNN bidirectional LSTM (TCN-BiLSTM) model was proposed to estimate joint angles and moments. A common benchmark dataset [25], including data from three IMUs during different locomotive activities, was used for the TCN-BiLSTM model training and testing. The joint angles and moments estimated by the TCN-BiLSTM model were compared with the reference joint angles and moments obtained from the motion capture system. Eventually, the performance of the proposed model was compared with the LSTM [26] artificial neural network (ANN) [23] and gated recurrent unit (GRU) [27].

## 2. Materials and Methods

### 2.1. Gait Dataset

A public Benchmark Dataset [25] was employed to test the proposed TCN-BiLSTM model. The dataset contained the IMU data collected from 22 healthy subjects in four different locomotion activities, as well as the kinematics and kinetics results derived from motion analyses. The locomotion activities included treadmill walking, level-ground walking, ramp ascent/descent, and stair ascent/descent. Data during treadmill walking included 28 different speeds ranging from 0.5 to 1.85 m/s in 0.05 m/s increments. Data during level-ground walking were collected at slow, normal, and fast speeds, relative to the preferred speed of the subjects. The mean walking speed for all subjects was 0.88 ± 0.19 m/s for slow walking, 1.17 ± 0.21 m/s for normal walking, and 1.45 ± 0.27 m/s for fast walking. Ramp ascent/descent trials were conducted at six different inclination angles. Similar to ramp trials, stair ascent/descent trials were performed at four different stair heights of a six-step staircase. In the present study, incorrect data in three subjects were excluded. Data from 12 healthy male and seven healthy female individuals (height: 1.52–1.80 m, weight: 52.2–96.2 kg, Age: 19–33 years old) were chosen for model training and testing. During the data collection, motion capture markers were placed on the human body according to the Helen Hayes marker set. Three six-axis IMUs (Yost, Portsmouth, OH, USA) were used to collect motion data. For the thigh, shank, and foot, the IMUs were placed on approximately 3/4 of the distal end for each segment on the right lower limb. The marker trajectory, center of pressure, and ground reaction force were collected during different locomotion tasks. The 3D angular acceleration and 3D angular velocity of the thigh, shank, and foot were collected with three IMUs, respectively. Joint angles and joint moments of the lower limb were calculated by OpenSim [28] according to the motion data and ground reaction force. After that, the joint moments of the lower limb during four different locomotion patterns were normalized to the participant’s body weight.

The sample size adopted in the present study was determined according to experience from previous studies. In the previous studies, data collected from ~10 subjects were typically used to train deep learning models for the estimation of joint angles and joint moments [29,30]. In order to maximize the performance of the proposed model, 19 valid subjects from a total of 22 subjects were chosen in the present study.

### 2.2. Data Preprocessing and Feature Extraction

**Figure 1:**
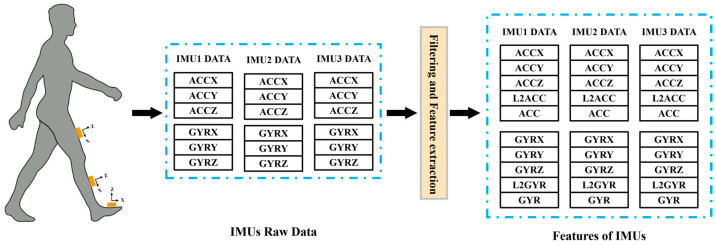
Block diagram of data processing and feature extraction.

Joint angles, joint moments, and raw data from three IMUs were split into different gait cycles based on the ground reaction force data of the instrumented leg. In total, 16,066 gait cycles for treadmill walking, 1798 gait cycles for level-ground walking, 3294 cycles for ramp ascent/descent, and 3063 cycles for stair ascent/descent were used in the present study. The segmented data were normalized using downsampling to ensure consistent data across different gait cycles. IMU data were filtered using a sixth-order Butterworth filter with a cutoff frequency of 100 Hz to remove the interference of environmental noise and soft tissue vibration. To improve the accuracy of the estimations, the *L*2 norm and average values of each gyroscope and accelerometer sensor data were extracted and integrated with 3-axis accelerometer and gyroscope data as features of IMU [31]. The data processing flow is summarized in Figure 1. The *L*2 norm and average values of *ACC* and *GYR* were computed according to (1) and (2):

(1) $$L2ACC=\sqrt{a_{x}^{2}+a_{y}^{2}+a_{z}^{2}}\;L2GYR=\sqrt{v_{x}^{2}+v_{y}^{2}+v_{z}^{2}}$$

(2) ACC=(ax+ay+az)3     GYR=(vx+vy+vz)3

where $a_{x}$, $a_{y}$, $a_{z}$ represent the 3-axis acceleration and $v_{x}$, $v_{y}$, $v_{z}$ represent the 3-axis angular velocity, respectively.

### 2.3. The Proposed TCN-BiLSTM Framework

**Figure 2:**
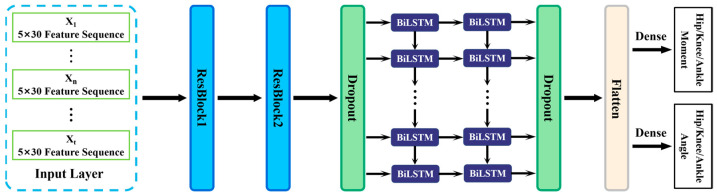
Architecture of the TCN-BiLSTM deep neural network.

The proposed TCN-BiLSTM model mainly contains TCN and BiLSTM modules and the architecture of the proposed model is shown in Figure 2. A Temporal Convolutional Neural Network (TCN) was designed to deal with time series data, using convolution’s powerful, robust characteristics to extract features across time steps, to better capture long-term dependencies in time series [32]. The BiLSTM contained forward LSTM and backward LSTM, and was able to deal with forward and backward time series data [33]. Due to the excellent performance of the TCN and BiLSTM in estimating time series data, a TCN-BiLSTM network was developed in this study for joint angle and joint moment regression.

Based on the spatio-temporal correlation of IMU data, a TCN-BiLSTM network was developed in this study for joint angle and moment estimation. Firstly, after filtering and feature selection, the IMU data were transformed to time series data Xt (shape = 5 × 30), which were utilized as input to the TCN-BiLSTM deep neural network. ‘Xt’ is a matrix of 5 × 30, referring to time slice data input to the deep learning model. The ‘5’ refers to the time steps. The ‘30’ represents the length of the feature data, which contain raw and artificial features of three IMUs. Secondly, the IMU data were fed into the residual block (filters = 8, kernel size = 1) with two dilated convolution layers. The residual block contained multiple layers that enhanced the depth of the model and alleviated the problem of gradient disappearance. The TCN block contained two residual blocks to better extract multivariate time series features with multiple time steps. The dropout layer (dropout = 0.5) was utilized to prevent the overfitting of the TCN. Dropout is a regularization method to reduce the overfitting of neural networks. Thirdly, the feature sequences output from the TCN were input into the BiLSTM layer, which contained eight cell units. The BiLSTM layer was used to extract temporal features from the data. Then the same dropout layer was again used to avert overfitting, avoiding gradient disappearance or gradient explosion. Fourthly, the flatten layer flattened the multi-dimensional features output from the BiLSTM layer to one dimension. Features output from the flatten layer were successively input to the dense layer (neurons = 1). Ultimately, the predictive regression task for joint moments and joint angles was completed in the output layer.

### 2.4. Temporal Convolutional Neural Network Module

**Figure 3:**
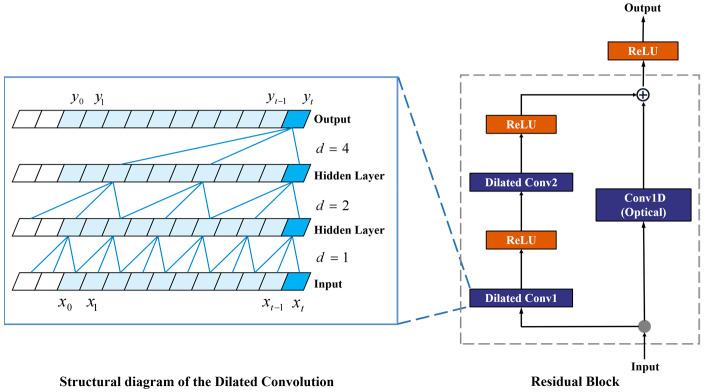
Architecture of the TCN block.

The Temporal Convolutional Neural Network (TCN) was initially proposed for processing the time-series data [32]. TCN is superior to long short-term memory (LSTM) neural networks and GRU in sequence modeling, and it can capture past information through a multi-layer architecture. TCN consists of a residual block and dilated convolution (Figure 3). The dilation factor (d) of the dilated convolution is 1, 2, and 4. The dilated convolution has a large receptive field that can cover all values from the input sequence. Dilated convolution allows interval sampling during convolution to obtain a larger effective sampling window to extract features across time steps. The residual block contains two dilated causal convolutions, a Conv1D and two ReLU activation functions. The residual block allows deep neural networks to be fully trained [34].

### 2.5. BiLSTM Module

**Figure 4:**
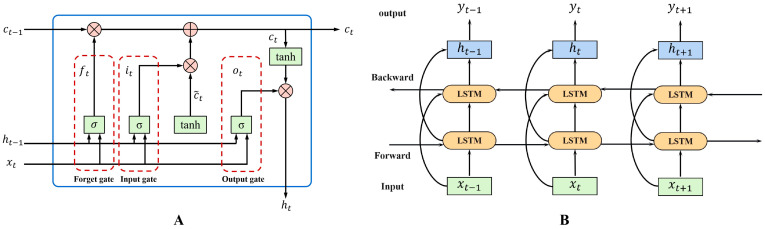
Architecture of the LSTM (**A**) and BiLSTM block (**B**).

LSTM introduces a memory cell to deal with long-term dependencies, and each LSTM unit is composed of three gates: forget gate, input gate, and output gate (Figure 4A) [35]. The LSTM controls the transmission state by gating the state, remembering what takes a long time to reflect, and forgetting the unimportant information. The forget gate determines what information is discarded from the cell state. The conclusion is made based on the state vector of $h_{t-1}$ and input vector $x_{t}$. The output of the forget gate is $f_{t}$, which has a value between 0 and 1, with 1 indicating full retention and 0 indicating full discard. The input gate determines the information to be stored in the cell state, where the sigmoid layer decides what values need to be updated and the tanh layer creates a new vector of candidate values and adds them to the LSTM memory. The output gate decides what information to output. The sigmoid layer is first used to obtain an initial output, then the tanh is used to scale the value to between −1 and 1 and then multiplied pair by pair with the output obtained from the sigmoid to obtain the output of the model. To extract the past and future features better, a BiLSTM deep neural network was introduced in this research, which contains both forward and backward LSTM (Figure 4B). Since BiLSTM can effectively solve the problem that the LSTM can only preserve the previous information, it is more conducive for time series regression.

### 2.6. Baseline Models

To evaluate the performance of the proposed TCN-BiLSTM model, LSTM [26], ANN [23], and GRU [27] were introduced as baseline models for comparison. The influence of data sources was removed by feeding the same training data into the deep learning models during four different locomotion modes.

LSTM model [26]: The LSTM model consists of two LSTM layers (each containing 50 LSTM cells) and a fully-connected layer. After that, the dense layer (neurons = 1) is used to predict joint moments and joint angles.

ANN model [23]: The ANN model consists of two fully-connected layers with 100 and 20 cells, respectively. The time series data is flattened after passing through the two fully-connected layers and then fed into the output layer to complete the prediction task.

GRU model [27]: The GRU model includes a GRU layer (units = 64) and a dropout layer. The dense layer (neurons = 1) is used to output the prediction results.

### 2.7. Dataset Segmentation Strategy

The proposed TCN-BiLSTM model was trained and tested using the leave-one-out-subject cross-validation approach. In detail, the public benchmark dataset was split into two parts: the test set and the training set. The test set included the data of one subject, and the training set was composed of the data of the other 18 subjects.

### 2.8. Evaluation Metrics

The Pearson correlation coefficient (*PCC*) and the root mean square error (*RMSE*) were introduced to evaluate the models’ performance. *PCC* represents the correlation between ground-truth, i.e., the joint angles/moments calculated by OpenSim, and estimated joint angles/moments. The *RMSE* indicates the square root of the deviation between estimated values and ground-truth. The *PCC* and *RMSE* can be calculated by using the following equations:

(3) $$PCC=\frac{cov(Y_{pre}{,\;Y}_{real})}{\sigma_{pre\;}\sigma_{real}}$$

(4) $$RMSE=\frac{1}{M}\sum {\left(Y_{pre}-Y_{real}\right)}^{2}$$

where $Y_{pre}$ is the estimated joint angles/moments, $Y_{real}$ is the real angles/moments, M is the number of samples, cov is the covariance, and σ is the standard deviation.

### 2.9. Implementation Details

Keras was used to train the TCN-BiLSTM model. During the feature extraction process, the window length was 5 and the sliding length was 1. Adam was employed as optimizer and the batch size was 64. The initial learning rate was 0.0001. Early stopping was used to achieve better generalization performance. Specifically, the training process was completed when the val_loss stopped decreasing by 20 epochs.

## 3. Results

**Figure 5:**
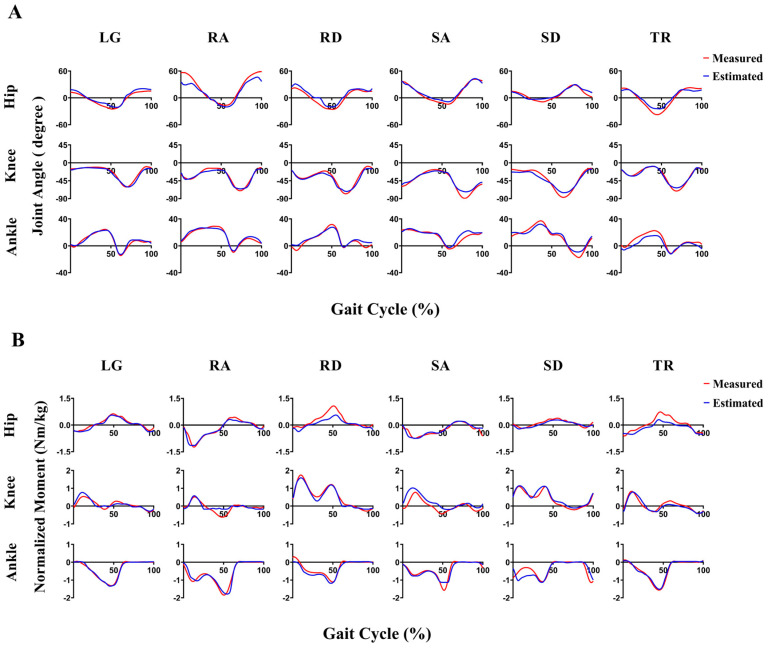
Comparisons between the actual joint angles/moments (red line) and estimated joint angles/moments (blue line) during one gait cycle for subject AB19. Subfigure (**A**) shows the comparison between the joint angle estimated by TCN-BiLSTM model and the actual joint angle. Subfigure (**B**) presents the comparison between the joint moment estimated by TCN-BiLSTM model and the actual joint moment. LG: level-ground walking, RA: ramp ascent, RD: ramp descent, SA: stair ascent, SD: stair descent, TR: treadmill walking.

The values of the average PCC of lower limb joint angles and moments obtained from the proposed TCN-BiLSTM neural network during four different movement modes were 0.92 and 0.87, respectively, which were highly correlated with the results obtained in the laboratory (Figure 5).

For the joint angles estimated using the proposed TCN-BiLSTM model, the highest estimation accuracy was achieved in treadmill walking mode; its average PCC was 0.92. Besides, the TCN-BiLSTM model obtained the same estimation accuracy in the level-ground walking and stair ascent/descent modes with an average PCC value of 0.92, higher than that of 0.90 for the ramp ascent/descent mode. Regarding lower limb joints, the average PCC of the hip, knee, and ankle joints were 0.91, 0.94, and 0.91, respectively (Table 1).

Regarding moment estimation, the TCN-BiLSTM model performed best in treadmill walking mode; the average PCC reached 0.93, but in level-ground walking mode, the average PCC was only 0.80. In the ramp ascent/descent and stair ascent/descent motion modes the average PCC results estimated by the TCN-BiLSTM model were 0.89 and 0.86, respectively. The average PCC of the TCN-BiLSTM model in estimating hip, knee, and ankle joint moments were 0.85, 0.85, and 0.91 respectively, and the mean PCC of the ankle joint with the highest estimation accuracy was just 6% higher than that of the hip and knee joints. The mean RMSE for the hip, knee, and ankle joints during the different movement patterns were 0.23 Nm/kg, 0.21 Nm/kg, and 0.23 Nm/kg, respectively (Table 2).

To compare the performance of the proposed TCN-BiLSTM model in estimating lower limb joint angles and moments, LSTM [26], ANN [23], and GRU [27] were trained and tested using the same dataset as in the present study. The comparison of the estimation accuracy of the above four deep learning neural networks is presented in Table 3 and Table 4. The comparison of the estimated joint angle/moment between the TCN-BiLSTM model and the baseline models during one gait cycle has been added to Appendix A. For lower limb joint angle and moment estimation, the average PCC derived from TCN-BiLSTM in treadmill walking mode is not significantly different from the other baseline models. However, the estimation accuracy of the TCN-BiLSTM model is significantly higher than that of LSTM, ANN, and GRU during the other three locomotion activities, such as level-ground walking, ramp ascent/descent, and stair ascent/descent.

## 4. Discussion

The present study proposed a subject independent TCN-BiLSTM deep learning model to estimate sagittal plane joint angles and moments of the lower limbs using three IMUs. The performance of the TCN-BiLSTM model was assessed during four locomotive activities. The mean PCC values of the hip/knee/ankle joint angles and moments derived from the proposed model were 0.91/0.94/0.91 and 0.85/0.85/0.91, respectively. Meanwhile, LSTM [26], ANN [23], and GRU [27] were introduced as baseline models to evaluate the estimation performance of TCN-BiLSTM. The comparison results suggested that the TCN-BiLSTM model outperforms LSTM [26], ANN [23], and GRU [27] in hip/knee/ankle joint angle and moment estimation. This work indicates that IMU combined with a deep learning model makes joint angle and moment estimation outside the laboratory possible. Compared with the traditional machine learning algorithms for single-task estimation, the TCN-BiLSTM model enhanced the robustness and applicability of multi-task estimation.

TCN has powerful feature extraction capabilities and is suitable for dealing with time series data. TCN has dilated convolution, which significantly increases the receptive field and allows it to better handle time series with long histories. The dilation convolution operation allows the output layer to observe the entire input data sequence even with a few network layers, thus reducing the required training parameters [36]. The residual network in TCN can avoid gradient decay and gradient disappearance during the training process [37]. The BiLSTM network is a variant of the LSTM network, which enables the given input data to be trained from both forward and backward directions. Applying the LSTM twice, forward and backward, can improve the long-term dependence of learning and thus improve model prediction accuracy [33]. In the present study, TCN and BiLSTM modules were used to compose the TCN-BiLSTM model, which integrates the feature extraction capability of the CNN and the time series data prediction capability of the LSTM to better extract temporal and spatial features from raw IMU data. 

The present results suggest that the accuracy of kinetic estimation is lower than that of kinematics, which indicates that the TCN-BiLSTM model might be more suitable for estimating joint angles. The knee joint angle estimation accuracy is the highest, with an average PCC of 0.94. Compared with the hip and ankle joints, the motion of the knee joint is mainly concentrated in the sagittal plane and its degree of freedom is relatively small. The movement of hip and ankle joints in the frontal and transverse planes may affect the estimation accuracy in the sagittal plane, resulting in a reduction in estimation accuracy [19,38]. In addition, the number and placement of IMUs also affect the estimation results [8,19]. The IMUs on the thigh and shank were attached adjacent to the knee joint. The main motion characteristics of the knee joint can therefore be captured, which allows the proposed model to derive better estimation accuracy in the knee joint angle estimation. Since the ankle joint has a smaller range of angles in the sagittal plane than the hip and knee joints, it obtains the minimum RMSE value.

The estimation accuracy of the ankle joint moment was higher than that of hip and knee joint moments. It was therefore suggested that the ankle joint moment might be easier to predict than the other joints for the proposed TCN-BiLSTM, while using three IMUs. Because the foot-attached IMU is located close to the ankle joint, it might be ideal for capturing the linear accelerations and angular velocities related to the ankle moment [39]. Typically, when estimating joint moments, level-ground walking was supposed to achieve higher prediction accuracy than ramp ascent/descent and stair ascent/descent due to the high repeatable motions and fixed mode of movement of gait compared with stairs/ramp. However, the estimation accuracy obtained by level-ground walking was the worst, which may be mainly due to the insufficient training data in the level-ground condition [40]. By contrast, the highest estimation accuracy was obtained from treadmill walking due to the large amount of training data and sufficient rhythm. The estimation accuracy during stair ascent/descent was lower than treadmill walking and ramp ascent/descent, possibly due to the different strategies in getting up and down stairs among subjects. This increased inter-subject variability in the stair data set might have made it difficult for the deep learning model to perform good estimations for those conditions. For example, some participants climbed upstairs with the soles of their forefeet while others climbed upstairs with their entire feet. The difference between individuals made it difficult for the deep learning model to estimate these conditions well, and led to the decline of estimation accuracy [29]. Overall, the average PCC of joint moment estimation for the hip/knee/ankle joints in different movement patterns was higher than 0.85, demonstrating the TCN-BiLSTM model’s application potential in multiple tasks.

During treadmill walking, the estimation accuracy of the TCN-BiLSTM model remained at the same level as the baseline models, which might be due to the high repeatability of the movement on the treadmill. By contrast, in complex locomotion activities, such as ramp ascent/descent and stair ascent/descent, the TCN-BiLSTM model demonstrated its superior feature extraction ability to adapt to the variability of motion, which dramatically improved the estimation accuracy than LSTM, ANN, and GRU models.

In general, the orientation of the IMU sensor in the global coordinate system and sensor-to-segment alignment need to be considered when using IMU-based gait analysis systems [10]. However, the alignment procedure between sensors and segments is prone to introduce alignment errors, and is the primary error source of the IMU-based gait analysis system [11]. To improve the estimation accuracy of IMU-based gait analysis systems, model-based filtering approaches, such as the Kalman filter, complementary filter, and gradient descent algorithm were proposed for gait analysis [41,42,43,44]. Nevertheless, the sensor-to-segment alignment is still a problem to be addressed. Of note, the deep learning models can learn the position and orientation of the IMUs, minimizing the calibration procedure while applying joint kinematic and kinetic estimation [17,45]. The TCN-BiLSTM deep neural network proposed in the present study does not require static calibration of the sensors, significantly improving its applicability in clinical settings and daily life scenarios.

Some deep learning algorithms have been developed previously for human gait analysis [46,47]. At the same time, some studies have only carried out a single kinematic or kinetic analysis using the real IMU data. The DeepConverLSTM model was proposed to predict the lower limb joint kinematics using five IMU sensors, achieving correlation results ranging from 0.70 to 0.89 [45]. In another previous work, a machine learning method was proposed to estimate the moments of the hip and knee joints based on raw IMU data derived from a mobile phone [39]. The mean absolute errors for the right and left hip joints were 36% and 29%, respectively. The estimation results indicated that more sensors or advanced models might be necessary to precisely analyze joint angles and moments. The simulated IMU data obtained from optical motion capture data were employed to estimate joint kinematics and kinetics using the standard feedforward neural network (FFNN) and LSTM model with mean correlation coefficients higher than 0.80 [16]. Likewise, a recent work used simulation methods to generate simulated IMU data combined with measured IMU data to augment the dataset to train the CNN model for the joint kinematic and kinetic prediction. When adding simulated IMU data, the root mean square error of joint angle and joint moment estimation were significantly reduced [48]. However, the simulated IMU data did not include factors that may introduce errors into the IMU data, such as noise caused by soft tissue movements, and individual differences between participants, which may have affected the calculation of joint angles and joint moments. Due to the gap between simulated and measured data, it is difficult to determine the performance of the mentioned methods in estimating joint angles and moments using measured IMU data. In this study, the proposed TCN-BiLSTM model implemented the estimation of hip, knee, and ankle joint angles and moments, which can provide more valuable information for gait analysis. In addition, the robustness and practicability in real-life scenarios of the TCN-BiLSTM model were enhanced by using measured IMU data from a wide range of motion conditions to train and test the model.

Furthermore, there are still a few limitations in the present study to be addressed in future studies. Firstly, though a relatively comprehensive dataset was used, which contained data on a wide range of walking conditions and walking speeds, the amount of data in some movement patterns was relatively small, such as level-ground walking. To address this limitation, the data from different public datasets can be merged to establish a larger dataset. Moreover, the data of individual subjects can also be augmented using the method of generative models [49]. The expanded dataset is capable of increasing the diversity of the dataset, allowing the deep learning models to better capture the variations between different subjects and the difference between different motion cycles of a single subject. Secondly, in-depth feature screening of the raw IMU data may enhance the estimation performance of the deep learning model. Feature processing can be performed in future deep learning models using the Ensemble Feature Score (EFS) and Profile Likelihood Maximization (PLM) algorithms [50] to improve prediction accuracy. Moreover, considering that the model may be deployed in wearable devices in the future, it may be necessary to solve the problem of the lightweight design of the prediction model based on knowledge distillation.

## 5. Conclusions

This study proposed a subject-independent TCN-BiLSTM deep learning model to estimate sagittal plane joint angles and moments under different walking conditions using three IMUs attached to the lower limb. Compared to the LSTM, ANN, and GRU, the TCN-BiLSTM model achieved higher accuracy in hip/knee/ankle joint angle and moment estimation. It therefore indicated the robustness and practicality of the novel established TCN-BiLSTM model. Moreover, the proposed model does not require the implementation of sensor-to-segment alignment and calibration procedures, which is more conducive to the timely estimation of joint angles and moments. Of note, in future studies, a larger sample size might be able to improve the reliability of the proposed model and derive better estimation accuracy. Meanwhile, the robustness of the proposed model could also be optimized to overcome the potential variations in kinematics between individuals and within different cycles. The present research enriches the application of IMUs and deep learning models to estimate joint angles and moments, which can provide a monitoring tool for rehabilitation process assessment and injury diagnosis.

## Figures and Tables

**Table 1:** Average (and standard error) of PCC and RMSE values of hip/knee/ankle joint angles estimated by TCN-BiLSTM in four different locomotion patterns.

	Hip Joint		Knee Joint		Ankle Joint	
	PCC	RMSE (°)	PCC	RMSE (°)	PCC	RMSE (°)
**Level Ground**	0.91 ± 0.08	9.47 ± 2.70	0.94 ± 0.06	7.75 ± 2.51	0.90 ± 0.05	4.36 ± 1.10
**Ramp**	0.89 ± 0.05	10.61 ± 2.89	0.92 ± 0.04	8.78 ± 2.29	0.89 ± 0.06	5.87 ± 1.08
**Stair**	0.90 ± 0.04	9.27 ± 2.93	0.95 ± 0.03	9.38 ± 2.13	0.92 ± 0.03	6.13 ± 1.00
**Treadmill**	0.95 ± 0.02	8.51 ± 2.71	0.95 ± 0.04	7.73 ± 2.14	0.91 ± 0.04	4.46 ± 0.92
**Mean**	0.91 ± 0.05	9.47 ± 2.81	0.94 ± 0.04	8.41 ± 2.27	0.91 ± 0.05	5.21 ± 1.03

**Table 2:** Average (and standard error) of RMSE and PCC values of hip/knee/ankle joint moments estimated by TCN-BiLSTM in four different locomotion patterns.

	Hip Joint		Knee Joint		Ankle Joint	
	PCC	RMSE (Nm/kg)	PCC	RMSE (Nm/kg)	PCC	RMSE (Nm/kg)
**Level Ground**	0.80 ± 0.14	0.22 ± 0.09	0.72 ± 0.14	0.22 ± 0.07	0.87 ± 0.09	0.27 ± 0.08
**Ramp**	0.84 ± 0.07	0.29 ± 0.06	0.90 ± 0.04	0.25 ± 0.06	0.93 ± 0.04	0.20 ± 0.04
**Stair**	0.80 ± 0.05	0.19 ± 0.02	0.89 ± 0.04	0.22 ± 0.05	0.88 ± 0.05	0.23 ± 0.04
**Treadmill**	0.94 ± 0.02	0.22 ± 0.06	0.90 ± 0.05	0.16 ± 0.04	0.94 ± 0.05	0.20 ± 0.06
**Mean**	0.85 ± 0.07	0.23 ± 0.06	0.85 ± 0.07	0.21 ± 0.06	0.91 ± 0.06	0.23 ± 0.06

**Table 3:** The average PCC derived from different deep learning models in joint angle estimation during different locomotion activities. *: *p* ≤ 0.05.

	LSTM	ANN	GRU	TCN-BiLSTM
**Level Ground**	0.90 *	0.90 *	0.85 *	0.91
**Ramp**	0.86 *	0.88	0.84 *	0.90
**Stair**	0.90 *	0.90 *	0.89 *	0.92
**Treadmill**	0.94	0.95 *	0.94	0.94

**Table 4:** The average PCC of different deep learning models in joint moment estimation during different locomotion activities. *: *p* ≤ 0.05.

	LSTM	ANN	GRU	TCN-BiLSTM
**Level Ground**	0.76 *	0.78 *	0.74 *	0.80
**Ramp**	0.82 *	0.81 *	0.81 *	0.89
**Stair**	0.84 *	0.81 *	0.82 *	0.86
**Treadmill**	0.92	0.93	0.92	0.93

**Figure A1:**
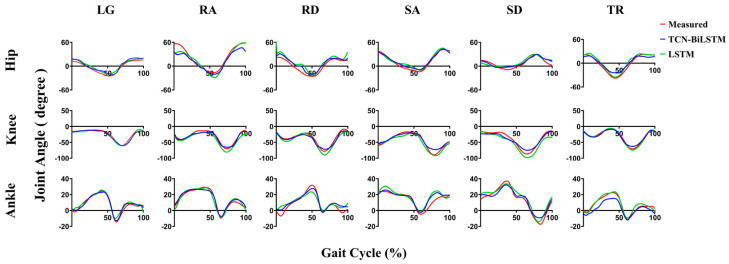
The joint angles estimated by the TCN-BiLSTM model (blue line) and LSTM model (green line) during one gait cycle for subject AB19. The red line represents the actual joint angle.

**Figure A2:**
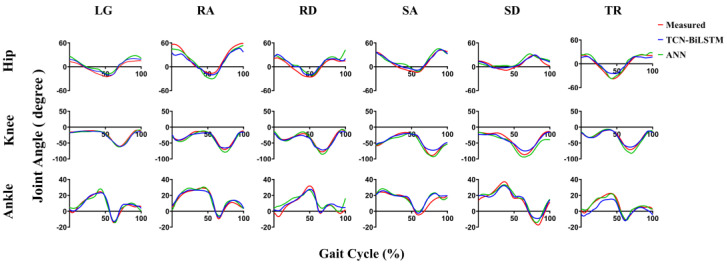
The joint angles estimated by the TCN-BiLSTM model (blue line) and ANN model (green line) during one gait cycle for subject AB19. The red line represents the actual joint angle.

**Figure A3:**
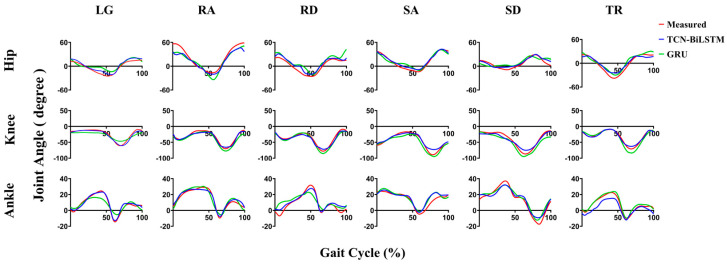
The joint angles estimated by the TCN-BiLSTM model (blue line) and GRU model (green line) during one gait cycle for subject AB19. The red line represents the actual joint angle.

**Figure A4:**
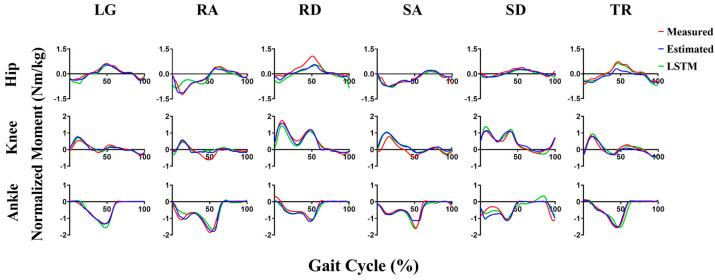
The joint moments estimated by the TCN-BiLSTM model (blue line) and LSTM model (green line) during one gait cycle for subject AB19. The red line represents the actual joint moment.

**Figure A5:**
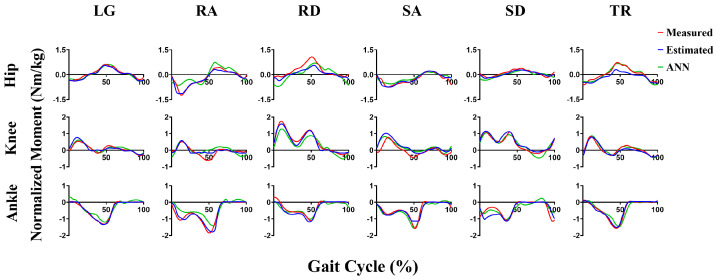
The joint moments estimated by the TCN-BiLSTM model (blue line) and ANN model (green line) during one gait cycle for subject AB19. The red line represents the actual joint moment.

**Figure A6:**
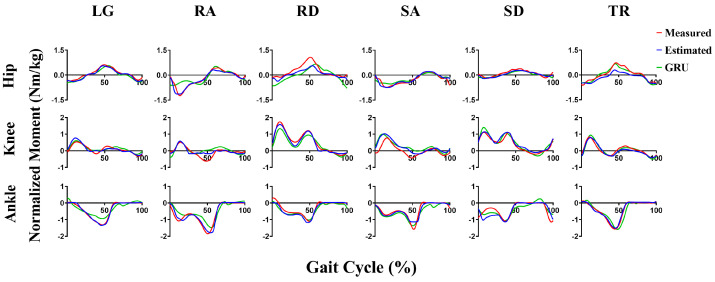
The joint moments estimated by the TCN-BiLSTM model (blue line) and GRU model (green line) during one gait cycle for subject AB19. The red line represents the actual joint moment.

## Data Availability

Data will be made available on request.
